# Leveraging a cuproptosis-based signature to predict the prognosis and drug sensitivity of cutaneous melanoma

**DOI:** 10.1186/s12967-023-03891-4

**Published:** 2023-01-30

**Authors:** Da Liu, Fan Yang, Tongtong Zhang, Rui Mao

**Affiliations:** 1 Department of Dermatology, Xiangya Hospital, Central South University, Changsha, China; 2 Emergency Department, Peking University Third Hospital, Peking University School of Medicine, Beijing, 100083 China; 3 The Center of Gastrointestinal and Minimally Invasive Surgery, The Third People’s Hospital of Chengdu, Chengdu, 610031 China; 4 Medical Research Center, The Third People’s Hospital of Chengdu, The Affiliated Hospital of Southwest Jiaotong University, The Second Chengdu Hospital Affiliated to Chongqing Medical University, Chengdu, 610031 Sichuan China

**Keywords:** Cutaneous melanoma, Cuproptosis-related genes, Immunotherapy, Prognosis, Nomogram

## Abstract

Immunotherapy is a vital treatment for patients with cutaneous melanoma (CM), but effective predictors to guide clinical immunotherapy are lacking. Cuproptosis is a newly discovered mode of cell death related to tumorigenesis. Exploring the relationship between the mode of cuproptosis and the effect of immunotherapy on CM could better guide clinical management. We clustered all patients with CM in the Cancer Genome Atlas (TCGA) database based on cuproptosis-related genes (CRGs). Prognosis, immunotherapeutic effect, tumor microenvironment score, expression of CD274, CTLA4, and PDCD1, and abundance of CD8 + T infiltration in group A were higher than in group B. Using a combination of LASSO and COX regression analysis, we identified 10 molecules significant to prognosis from differentially expressed genes between the two groups and constructed a cuproptosis-related scoring system (CRSS). Compared with the American Joint Committee on Cancer (AJCC) staging system, CRSS more accurately stratified CM patient risk and guided immunotherapy. CRSS successfully stratified risk and predicted the effect of immunotherapy in 869 patients with eight CM immunotherapy datasets and multiple other tumor immunotherapy cohorts. The nomogram model, which combined AJCC stage and CRSS, greatly improved the ability and accuracy of prognosis prediction. In general, our cuproptosis-related scoring system and nomogram model accurately stratified risk in CM patients and effectively predicted prognosis and the effect of immunotherapy in CM patients.

**Supplementary Information:**

The online version contains supplementary material available at 10.1186/s12967-023-03891-4.

## Introduction

Cutaneous melanoma (CM) is a highly malignant skin cancer that originates from melanocytes. Over the past few decades, its incidence among white people has increased dramatically, with 23,0000 new cases worldwide each year (World Health Organization) [1]. Immunotherapy is an indispensable and important treatment for CM [2]. In recent years, CM immunotherapy has made exciting progress and ushered in a new era for CM therapy. Compared with conventional chemotherapy, immunotherapy can cause an unprecedented, sustained response in patients with advanced cancer. However, this reaction occurs only in a relatively small number of patients, and the effect varies greatly among CM patients. These clinical challenges drive researchers to identify new tools to predict which patients are inherently resistant to targeted therapy and immunotherapy. This can better guide clinical management of patients and promote the rational use of clinical resources.

Copper is a mineral nutrient that is involved in cell proliferation and death pathways [3]. The link between copper and cancer has been well established, and many studies have shown that tumors require higher levels of copper than healthy tissues. Cancer cells also have a higher demand for copper than non-mitotic cells. It has been reported that the concentration of copper in tumors and serum is increased in both animal models and patients with breast, lung, gastrointestinal, oral, thyroid, gallbladder, gynecologic, and prostate cancer [4, 5]. Copper can also promote tumor angiogenesis and lead to tumorigenesis, growth, and metastasis. Cuproptosis is a mode of cell death induced by copper ions that is dependent on the accumulation of copper ions. When the cellular concentration of copper ions reaches a certain level, they regulate cell death through targeting acylated proteins in the TCA cycle [6]. CRGs affect tumorigenesis, invasion, and metastasis in a manner similar to ferroptosis and pyroptosis genes. cuproptosis is closely related to the progression of cancer and is a potential new therapeutic target for the targeted killing of cancer cells [4, 7].

The interaction between immune regulation and tumor cells in the tumor microenvironment (TME) can modulate the effect of immunotherapy. The TME plays an important role in these interactions via suppressing or enhancing the immune response. Emerging evidence suggests that copper overload and cuproptosis lead to immune dysfunction through the creation of reactive oxygen species (ROS). For example, ROS may contribute to the release of damage-related molecular patterns (DAMP), significantly regulating the immune response [8, 9]. Therefore, the molecular characteristics of CRGs may provide important insight into the characteristics of the TME and the potential mechanism of CM. Delineation of cuproptosis patterns and their inherent mutation patterns, immunotherapeutic effects, TME differences, and prognostic effects in patients with CM will provide new insights into the mechanisms of occurrence, development, and potential effects of immunotherapy on CM.

In this study, we systematically studied the expression of CRGs, somatic mutations, and CNV patterns in patients with CM using CM data from the TCGA and GEO databases. Two different modes of cuproptosis were identified in patients with CM, and differences in the TME and immunotherapy between the two modes were compared. To better guide clinical management, we constructed a CRSS and applied it to eight immunotherapy cohorts containing 502 CM patients. Results showed good risk stratification and prediction of the effects of immunotherapy. Finally, we combined four clinical features (age, sex, AJCC stage, CRSS) to construct a prognostic model. Results showed that the prognostic capability and stability of the combined model was greatly improved compared to each single variable alone.

## Methods

### Data collection and preprocessing

The download and processing of the CM dataset from the TCGA and GEO databases is described in our previous study [10]. The normal human skin transcriptome data stored in the GTEx database were downloaded from the UCSC Xena database [11] (http://xena.ucsc.edu/) (Additional file 1: Table S1) and used as a control (TPM format). Eleven immunotherapy datasets including phs000452, PRJEB23709, Nathanson_2017, GBM-PRJNA482620, GSE91061, nonsqNSCLC-GSE93157, GSE93157, GSE100797, GSE78220, GSE106128, and Braun_2020 were downloaded from the Tumor Immunotherapy Gene Expression Resource (TIGER) database. This is a web-accessible portal for integrative analysis of gene expression data related to tumor immunology (Additional file 1: Table S2). TIGER contains bulk transcriptome data for 1508 tumor samples with immunotherapy clinical outcomes [12].

### Tumor somatic mutation and copy number variant analysis

The catastrophic landscape of TCGA-SKCM patients was calculated and visualized using the “maftool” package. Missense, silence, nonsense, frameshift/in-frame insertions and deletions, and uninterruptions were counted; synonymous mutations were not counted [13]. The TMB score was calculated using the total number of somatic mutations. CNV analysis was carried out through GISTIC_2.0, and the CNV and somatic mutation results were integrated into the waterfall map using the “mclust” and “NMF” packages.

### Protein–protein interaction (PPI) and enrichment analysis

The PPI network of 10 CRGs was analyzed and visualized using GENEMANIA tools [14]. GO-BP enrichment analysis and gene set enrichment analysis (GSEA) were performed via the clusterProfiler [15] package. The input file included the expression matrix and grouped text of the log2 transformation. The P value was adjusted using the Benjamini–Hochberg (BH) method, and the threshold of the adjusted P value was set to 0.05.

### Unsupervised clustering and differential analysis

Based on the expression of 10 CRGs, we used the ConsensuClusterPlus package [16] to perform unsupervised cluster analysis of 469 CM patients. The number and stability of clusters were determined using the consensus clustering algorithm [17]. This process was repeated 1000 times to ensure the stability of the classification. We used the R software limma [18] package to analyze differentially expressed genes between the two groups. We screened genes with significant differences between the two groups using an adjusted P < 0.05 and|log2FC (fold change)|> 1.

### TIDE analysis and cancer drug sensitivity analysis

We used Tumor Immune Dysfunction and Exclusion (TIDE, http://tide.dfci.harvard.edu/) [19] analysis to study the differences in response to immune checkpoint inhibitor therapy in different CM patient groups. CM patients with a TIDE score greater than 0 were defined as non-responders to immune checkpoint inhibitor therapy, where patients with a TIDE score less than 0 were defined as responders [19]. In addition, we compared the difference in Dysfunction and Exclusion scores between the two groups.

### Analysis of immune score and immune cell infiltration

We used the ESTIMATE [20] website to calculate the immune score and stromal score of each CM patient in the TCGA-SKCM cohort. We used the Wilcoxon test [21] to further analyze the differences in immune score and stromal score between different groups. ESTIMATE score is a combination of stromal score and immune score, and can be used as an index to evaluate the purity of a tumor [20]. Based on the TCGA-SKCM cohort matrix, we calculated the immune cell abundance of each patient using CIBERSORT [22], MCP Counter [23], TIMER (http://timer.comp-genomics.org/) [24], and GSVA, among other software packages. We excluded samples with a P > 0.05. Finally, the differences in immune cell subtypes among different groups were analyzed via the Mann–Whitney *U* test.

### Construction of the cuproptosis-related scoring system

Based on survival data and using the R software lasso package, we constructed the CRSS for differentially expressed genes between the two groups via univariate COX regression analysis, lasso regression analysis, and multivariate COX regression analysis. The risk score of each patient in the training group was calculated using the Cox proportional hazards model (PH model): ${\widehat{h}}_{i}\left(t\right)={\widehat{h}}_{0}\left(t\right)exp\left({x}_{i}^{^{\prime}}\widehat{\beta }\right)$ (where exp is the prognostic gene expression level, β is the multivariate COX regression model regression coefficient, and ${h}_{0}\left(t\right)$ is the baseline hazard function.) [10] All CM samples were divided into a high-risk-score group and a low-risk-score group based on the median risk score. We used the Kaplan–Meier method to perform survival analysis, and the log-rank test was used to compare survival between groups. Using the R software package “SurvivalROC,” we drew the receiver operating characteristic curve (ROC) and calculated the corresponding area under the curve (AUC).

### Construction of the prognostic nomogram

We first performed univariate and multivariate Cox regression analyses of CRSS and other clinicopathological factors to evaluate whether CRSS is an independent risk factor for CM. Then, based on the R packages “RMS,” “Hmisc,” “Latge,” “Formula,” and “Foreign,” the nomogram model was constructed using the indices in the multivariate Cox regression model. We used the “nomogramEx” package to extract the scoring function of each variable in the model and simulate the formula for calculating survival probability. We also used the “DynNom” package to validate the nomogram model results. The total risk score was calculated according to each predictive factor in the nomogram; the median of the total risk score was taken as the cutoff value [25, 26]. We then divided the patients into a high-risk-score group and a low-risk-score group. The prediction capability and accuracy of the nomogram was validated by C index, ROC analysis, and calibration curve. We used a similar analysis process for the validation cohorts. Finally, by quantifying the net benefit under different threshold probabilities in the cohort [27], the clinical validity of the nomogram was determined using a clinical decision curve analysis (R packages “rms” and “rmda”) [28].

### Statistical analysis

Two or more continuous variables consistent with a normal distribution were analyzed by the *t*-test or analysis of variance (ANOVA), respectively. The Mann–Whitney *U* test and the Kruskal–Wallis test were used for two or more continuous variables that did not conform to the normal distribution. P values were corrected using the BH method [29]. An adjusted P‐value < 0.05 was considered statistically significant.

## Results

### Genetic characteristics and transcriptional changes in cuproptosis-related genes in CM

Additional file 1: Fig. S1 shows the entire analytic process of the study.

**Fig. 1:**
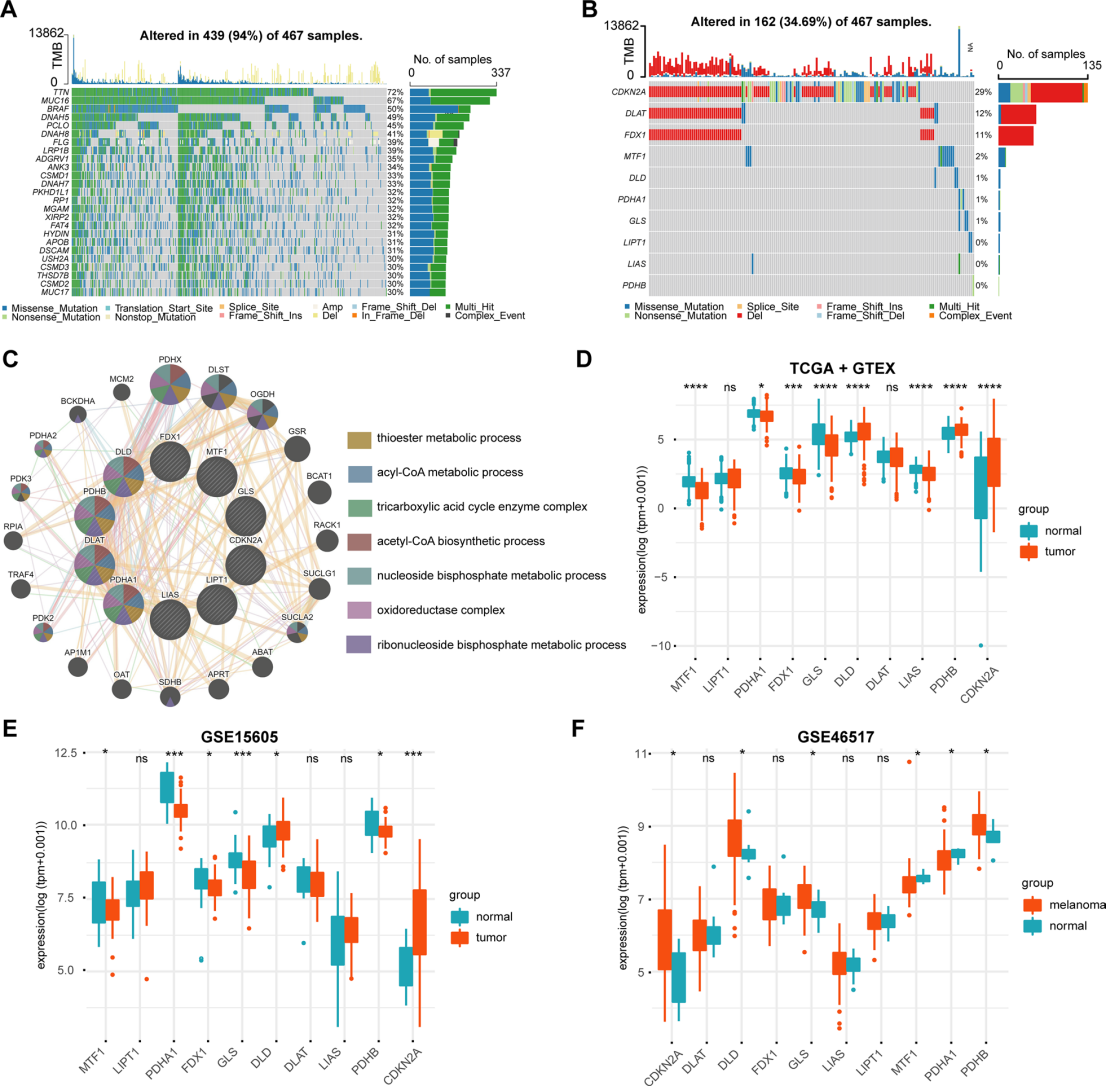
Genetic characteristics and transcriptional changes of cuproptosis related genes in CM. **A**. Somatic mutation and CNV landscape of all patients in TCGA-SKCM data set. **B**. Somatic mutation and CNV landscape of 10 CRGs in all patients in TCGA-SKCM dataset. **C**. Network diagram of PPI analysis and enrichment analysis of 10 CRGs. **D**. Differential expression of 10 CRGs in TCGA and GTEx datasets between CM patients and normal skin tissues. **E**, **F**. Differential expression of 10 CRGs in GSE15605 (**E**) and GSE46517 (**F**) datasets between CM patients and normal skin tissues. *: P < 0.05; **: P < 0.01; ***: P < 0.001; ****: P < 0.0001

Data processing of the TCGA and GEO data sets, as well as merging and removing of batch effects from the GEO datasets were carried out as described in our previous study (10.1111/cas.15499, Additional file 1: Fig. S1). First, we analyzed the somatic mutation and CNV landscape of 469 CM patients using the TCGA-SKCM dataset (Fig. 1A). Somatic mutations or CNVs were found in 439 of the 467 CM samples (94%). Next, we analyzed the somatic mutations and CNV patterns of 10 CRGs from CM patients (Fig. 1B). Somatic mutations or CNVs were found in the CRGs of 162 out of 467 CM samples (34.69%); these were mainly caused by chromosome deletion variation. Chromosome deletions were mainly found in CDKN2A, DLAT, and FDX1, where somatic mutations mainly occurred in CDKN2A. In addition, through protein–protein interaction (PPI) analysis using the GeneMANIA website, we found that PDHB, DLAT, PDHA1, LIAS, and DLD were hub genes in the interaction network (Fig. 1C). We then compared the expression levels of 10 CRG s in CM and normal samples from the TCGA + GTEx, GSE15605, and GSE46517 data sets (Fig. 1D–F). The results suggested that, compared with normal tissues, the DLD, PDHB, and CDKN2A genes were highly expressed in CM samples. MTF1, PDHA1, FDX1, GLS, and LIAS were downregulated in patients with CM.

### Identification of cuproptosis subgroups in CM and comparison of prognosis and TME between the two groups

**Fig. 2:**
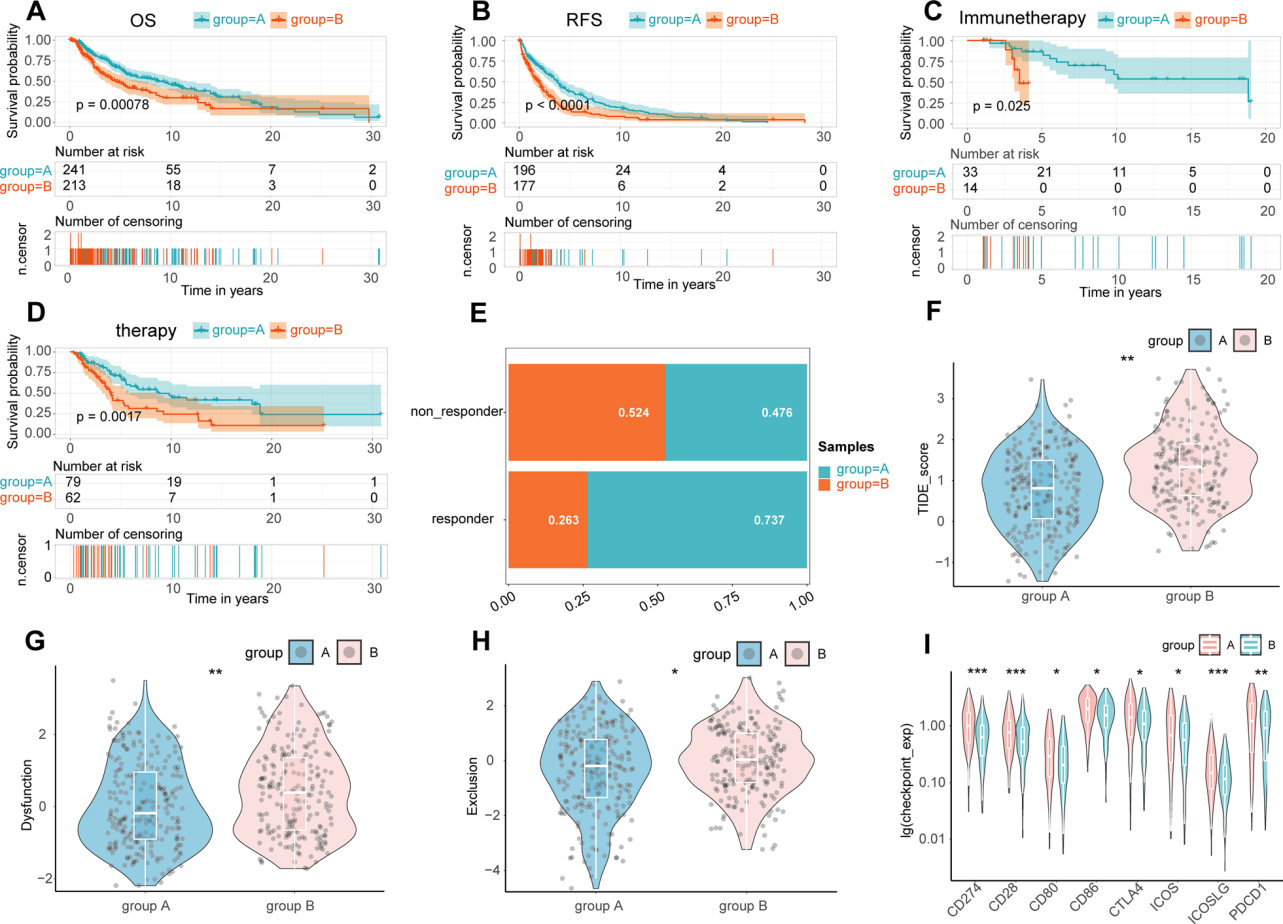
The effect and prognosis of immunotherapy in group A were significantly better than those in group B. The OS **(A)** and RFS **(B)** of CM patients in group A were significantly better than those in group B. The effect of immunotherapy (**C**) and chemotherapy (**D**) in group A was significantly better than that in group B in CM. **E**. Comparison of the proportion of patients responding to immunotherapy between the two groups. Comparison of TIDE (**F**), Dysfunction (**G**), and Exclusion (**H**) scores between the two groups. **I**. Comparison of the expression of 8 common immune checkpoints between the two groups. *: P < 0.05; **: P < 0.01; ***: P < 0.001; ****: P < 0.0001

The consistency of cluster analysis is dependent on determination of the K value. We used the K value of the cumulative distribution function (CDF) with a small slope of decline. According to our results, when K was equal to 2, the consistency was the best. Therefore, based on unsupervised clustering, we successfully divided the TCGA-SKCM data into two groups (Additional file 1: Fig. S2A–C). Interestingly, KM analysis suggested that the overall survival (OS) and recurrence free survival (RFS) of group A were significantly better than group B (log‐rank test P‐value < 0.001, Fig. 2 A, B). In addition, the effects of immunotherapy and chemotherapy in group A were significantly better than in group B (log‐rank test P‐value < 0.05, Fig. 2C, D). TIDE analysis results of all TCGA-SKCM samples showed that the TIDE score in group A was significantly lower than in group B, and the immunotherapy response rate of group A was significantly higher than group B (Fig. 2E, F). Further, the Dysfunction and Exclusion scores in group A were significantly lower than in group B (Fig. 2G, H). Results of the immune checkpoint analysis showed that the expression of CD274, PDCD1, and CTLA4 in group A was significantly higher than in group B (Fig. 2I) . The above results suggest that the sensitivity and effects of immunotherapy in group A were significantly better than in group B.

**Fig. 3:**
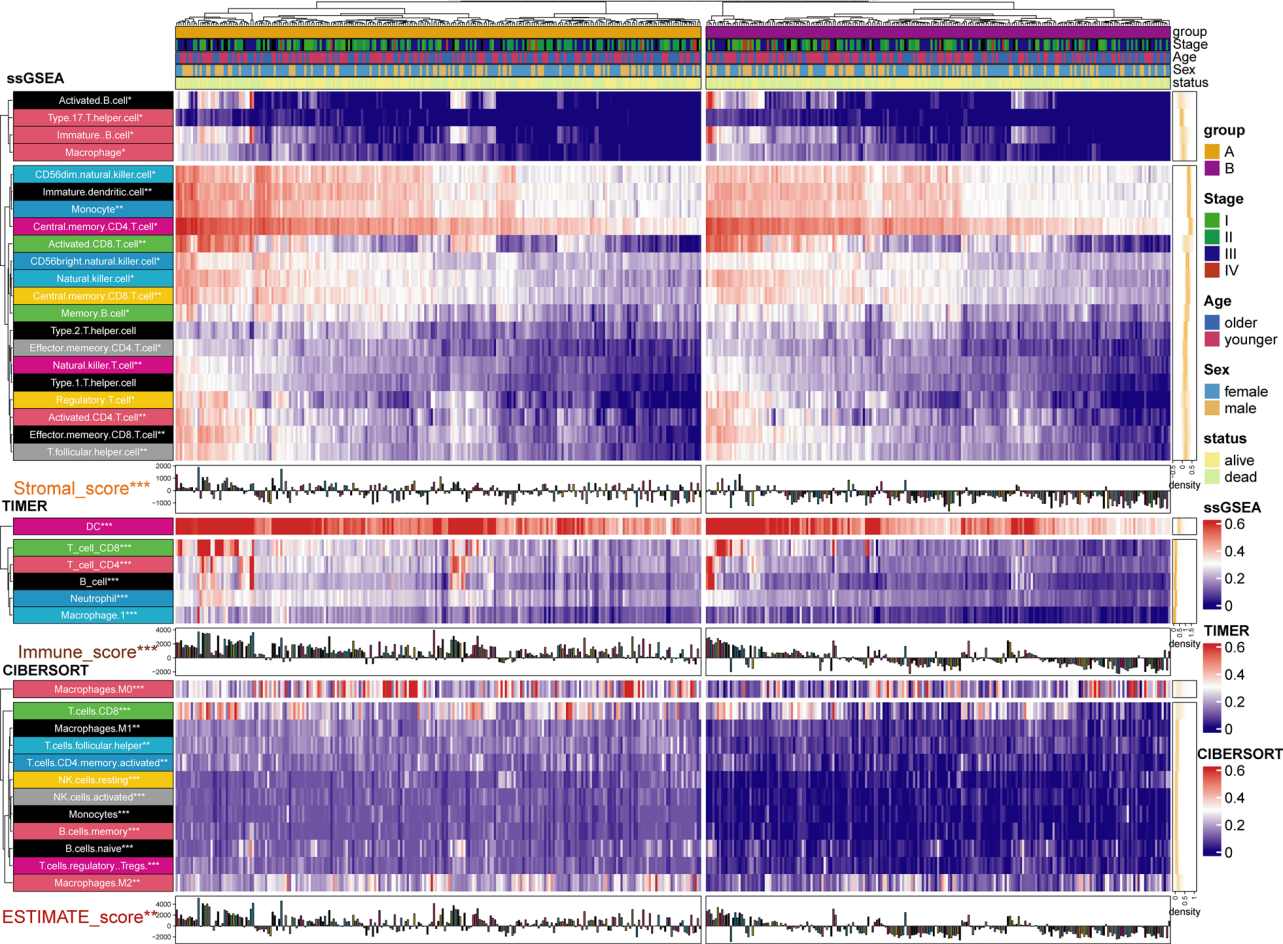
Difference of immune score and immune cell infiltration between group A and group B. The results were shown in the form of a complex heat map, in which the abundance of immune cell infiltration predicted by ssGSEA, TIMER, and CIBERSORT between the two groups was presented in the form of a heatmap. The immune score, stromal score, and ESTIMATE score predicted by ESTIMATE are displayed in the form of a bar chart. The yellow density chart on the right side of the heat map shows the average of each index. The statistical method of Mann- WhitneyU test was used to compare the two groups. *: P < 0.05; **: P < 0.01; ***: P < 0.001; ****: P < 0.0001

Tumor mutation load (TMB) and TME are vital biomarkers for the prediction of immunotherapy efficacy in CM. TMB has attracted much attention in immunotherapy. In addition, TMB and PD-L1 are important biomarkers for the prediction of PD-1 antibody efficacy [30]. Hodi et al. [31] showed that in patients receiving the anti-PD-1 inhibitor Nivolumab (NIVO) or NIVO combined with the anti-CTLA-4 inhibitor ipilimumab (IPI) or IPI alone, high (> median) TMB predicted longer survival than low (≤ median) TMB. Based on somatic mutation data from the TCGA-SKCM samples, we calculated and compared TMB between the two groups. The results showed that the TMB in group A was significantly higher than in group B (Additional file 1: Fig. S2D). To further investigate the difference in TME between the two groups, we evaluated the immune score of both groups and estimated the abundance of immune cell infiltration using four methods. The results from the ESTIMATE package calculation showed that the immune score, matrix score, and ESTIMATE score of group A were significantly higher than in group B (Additional file 1: Fig. S3A). Using a median cutoff value, the overall survival rate of CM patients with a high immune score and ESTIMATE score was significantly higher than CM patients with a low immune score (Additional file 1: Fig. S3B–D). The aggregation of CD4 + T cells and CD8 + T cells enhanced immune capacity and anti-tumor activity in CM [32, 33]. NK cells are a type of lymphocyte that possess cytotoxic activity and can effectively respond to the presence of a variety of tumor cells [34]. A decrease in the number of NK cells and CD8 + T cells has been related to adverse outcomes [35]. Using four methods to predict the abundance of immune cell infiltration (MCP, CIBERSORT, TIMER, and ssGSEA) we showed that the abundance of CD8 positive T cells, natural killer T cells, and macrophages in group A was significantly higher than in group B (Fig. 3 and Additional file 1: Fig. S2D). The above results further confirmed that the immunotherapy response and immune activity in group A was better than in group B.

### The function of group a was mainly concentrated in pathways related to immunity and immunotherapy

First, we performed differential expression analysis between group A and group B. Results identified a total of 2207 differentially expressed genes. Among the 10 CRGs, CDKN2A and DLD were significantly downregulated and DLAT, FDX1, and LIPT1 were significantly upregulated in group A (Additional file 1: Fig. S2D). Then, we performed GSEA on CM samples using the four enrichment analysis databases. Results from GSEA-GOBP and GSEA-KEGG suggested that the functional enrichment of group A tended toward pathways for immunity and immunotherapy such as B cell mediated immunity, lymphocyte mediated immunity, immunoglobulin mediated immune response, PD-L1 expression, PD-1 checkpoint pathway in cancer, Th1 and Th2 cell differentiation, and Th17 cell differentiation (Additional file 1: Fig. S4A, B). The GSEA-reactomePA and GSEA-hallmarker results suggested that the enriched pathways in group A tended toward immunotherapy such as PD-1 signaling, HALLMARK_IL6_JAK_STAT3_SIGNALING, HALLMARK_INTERFERON_GAMMA_RESPONSE, HALLMARK_TNFA_SIGNALING_VIA_NFKB and HALLMARK_IL6_JAK_STAT3_SIGNALING. In addition, cell cycle-related pathways, such as Cyclin A/B1/B2-associated events during G2/M transition, G1 Phase, Oncogene Induced Senescence, Senescence-Associated Secretory Phenotype (SASP), p53-Dependent G1/S DNA damage checkpoint, and HALLMARK_E2F_TARGETS were enriched in group B (Additional file 1: Fig. S4C, D). These results suggest that group A had higher immune activity where group B was more inclined toward a senescence-related secretory phenotype (SASP) caused by cell cycle senescence. Interestingly, SASP can promote cancer progression and resistance to immunotherapy.

### Construction of the CRSS for CM

**Fig. 4:**
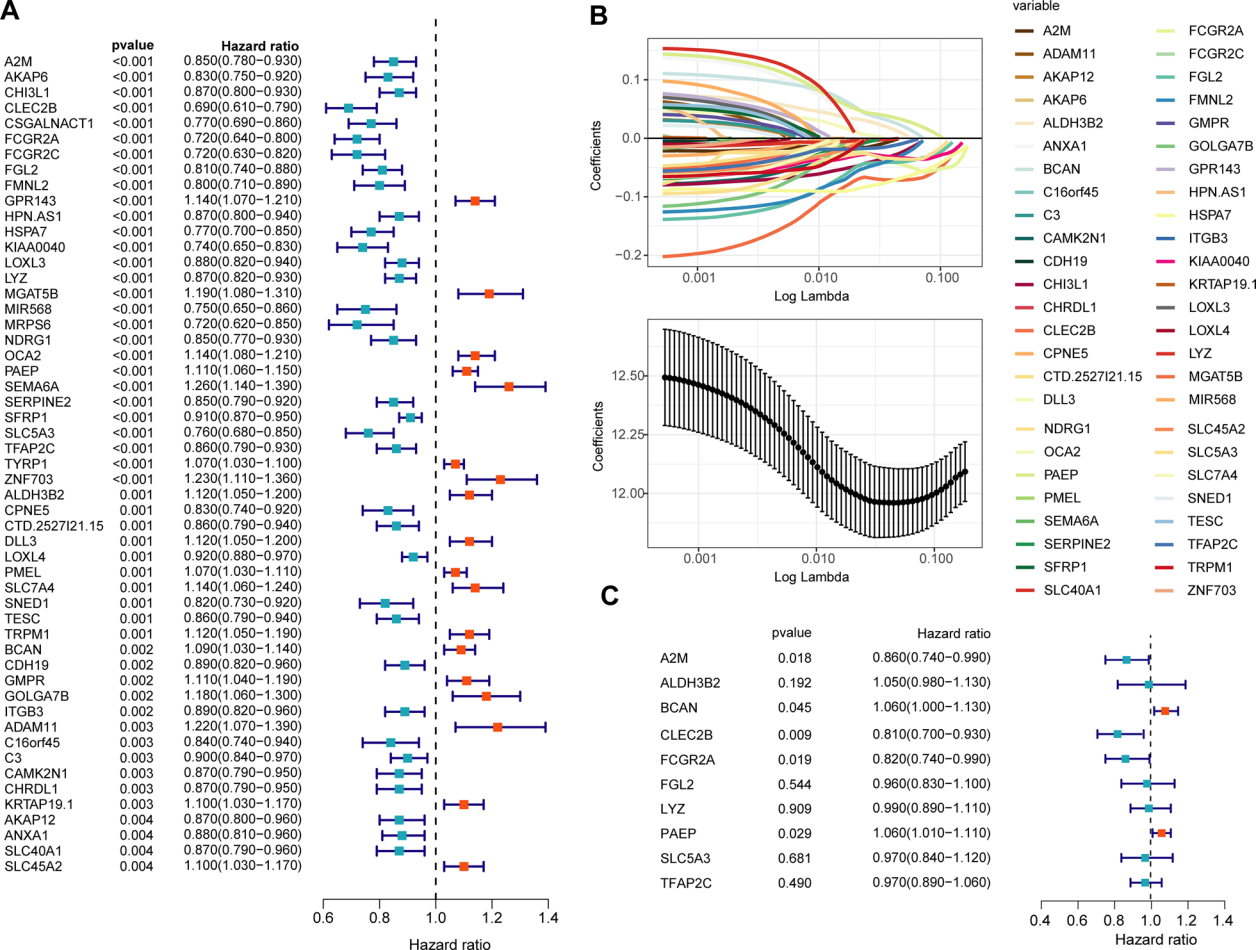
Construction of the CRSS for CM. A total of 2207 CRGs were evaluated by univariate Cox survival analysis, and 53 CRGs with P < 0.005 were filtered out and included in subsequent analyses (**A**). As shown in panel **B**, LASSO regression analysis identified 10 SRGs (lambda value = 10) that were subjected to multivariate Cox regression analysis (**C**)

First, we performed univariate COX regression analysis on 2207 differentially expressed genes between the two groups (Fig. 4A). Next, we included 53 genes with a P value of less than 0.005 to perform the next lasso regression analysis (Fig. 4B). Based on the standard lambda.1se (lambda value = 10), we identified 10 molecules for the construction of the CRSS. Then, we carried out multivariate COX regression analysis of the 10 molecules (Fig. 4C) and constructed the CRSS according to the multivariate COX regression analysis coefficient and expression of each variable. Taking the median cuproptosis-related risk score as the cutoff value, we divided the CM patients in the training and validation cohorts into high and low cuproptosis-related risk assessment groups (Additional file 1: Fig. S5A–F). We identified a significant difference in the expression of 10 CRGs between the two groups (Additional file 1: Fig. S5C–F). We further performed KM and ROC analysis in the training, validation, and test cohorts to test the ability of CRSS to predict the prognosis of patients with CM. In the training cohort, the OS and RFS of CM patients with a high cuproptosis-related risk score (CRRS) were significantly lower than those with a low CRRS (log‐rank test P‐value < 0.05, Additional file 1: Fig. S6A–C). In addition, the ROC analysis results suggested that the areas under curve of CRSS for predicting 5 year OS and RFS in CM patients were 0.76 and 0.78, respectively (Additional file 1: Fig. S6B–D). CRSS could also stratify the risk of CM patients and accurately predict OS, disease metastasis free survival (DMFS), and disease special survival (DSS) in the validation and test cohorts (log‐rank test P‐value < 0.0001, Additional file 1: Fig. S6E, G, I). The AUC of CRSS for predicting 5 year OS and DMFS in CM patients were 0.83 and 0.79 in the validation and test cohorts, respectively (Additional file 1: Fig. S6F, H).

### CRSS accurately predicted the effect of immunotherapy

**Fig. 5:**
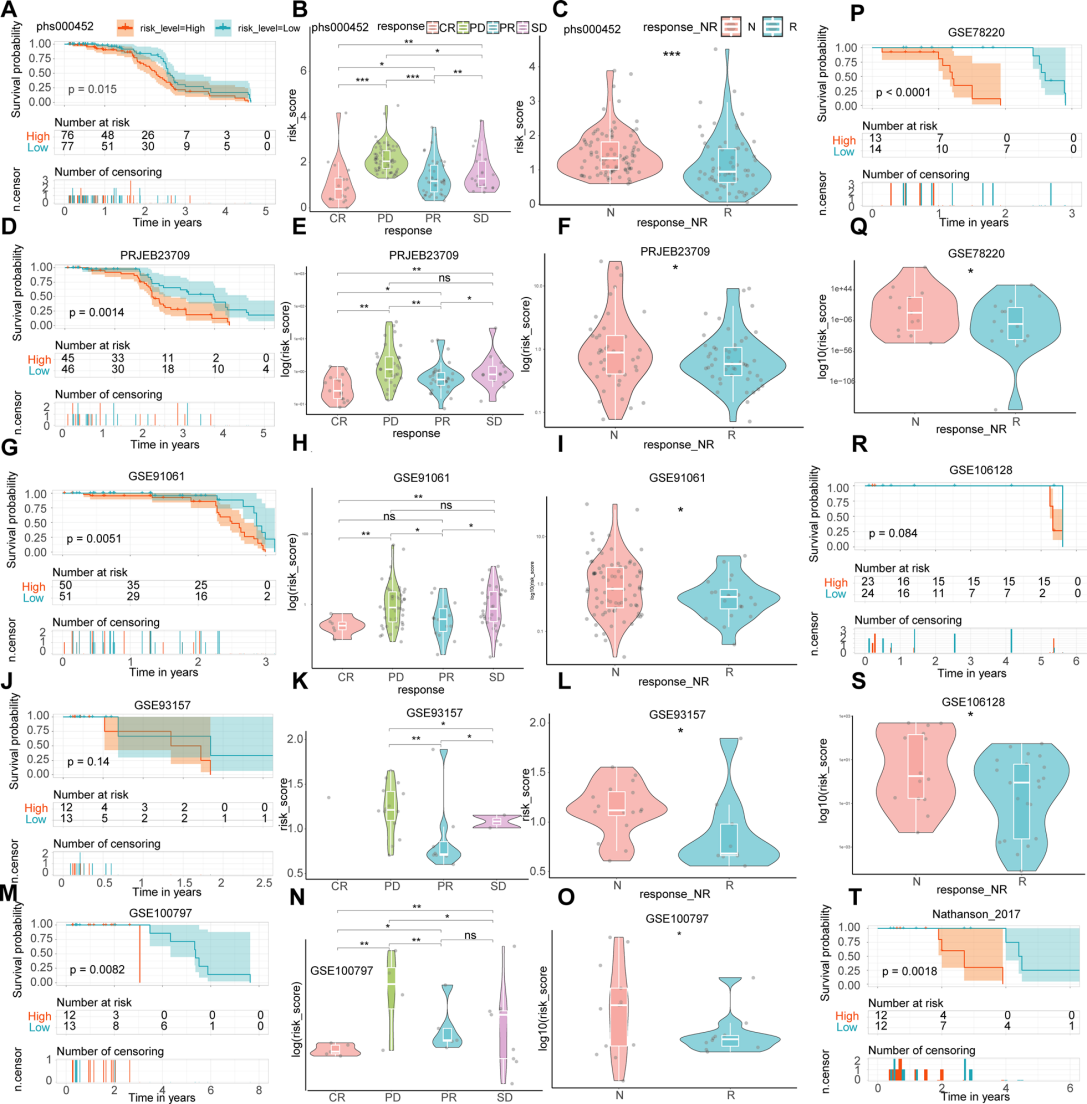
Validate the ability of CRSS to predict the effect of immunotherapy in other CM immunotherapy cohorts. KM analysis was used to compare the difference of immunotherapy effect between high and low CRRS groups in phs000452 (**A**), PRJEB23709 (**D**), GSE91061 (**G**), GSE93157 (**J**), GSE100797 (**M**), GSE78220 (**P**), GSE106128 (**R**), and Nathanson_2017 (**T**) dataset. Comparison of CRRS of different CM patients in different remission states after immunotherapy in phs000452 (**B**), PRJEB23709 (**E**), GSE91061 (**H**), GSE93157 (**K**), and GSE100797 (**N**) dataset. Comparison of CRRS of different CM patients in different response states after immunotherapy in phs000452 (**C**), PRJEB23709 (**F**), GSE91061 (**I**), GSE93157 (**L**), GSE100797 (**O**), GSE78220 (**Q**), and GSE106128 (**S**) dataset. *: P < 0.05; **: P < 0.01; ***: P < 0.001; ****: P < 0.0001

**Fig. 6:**
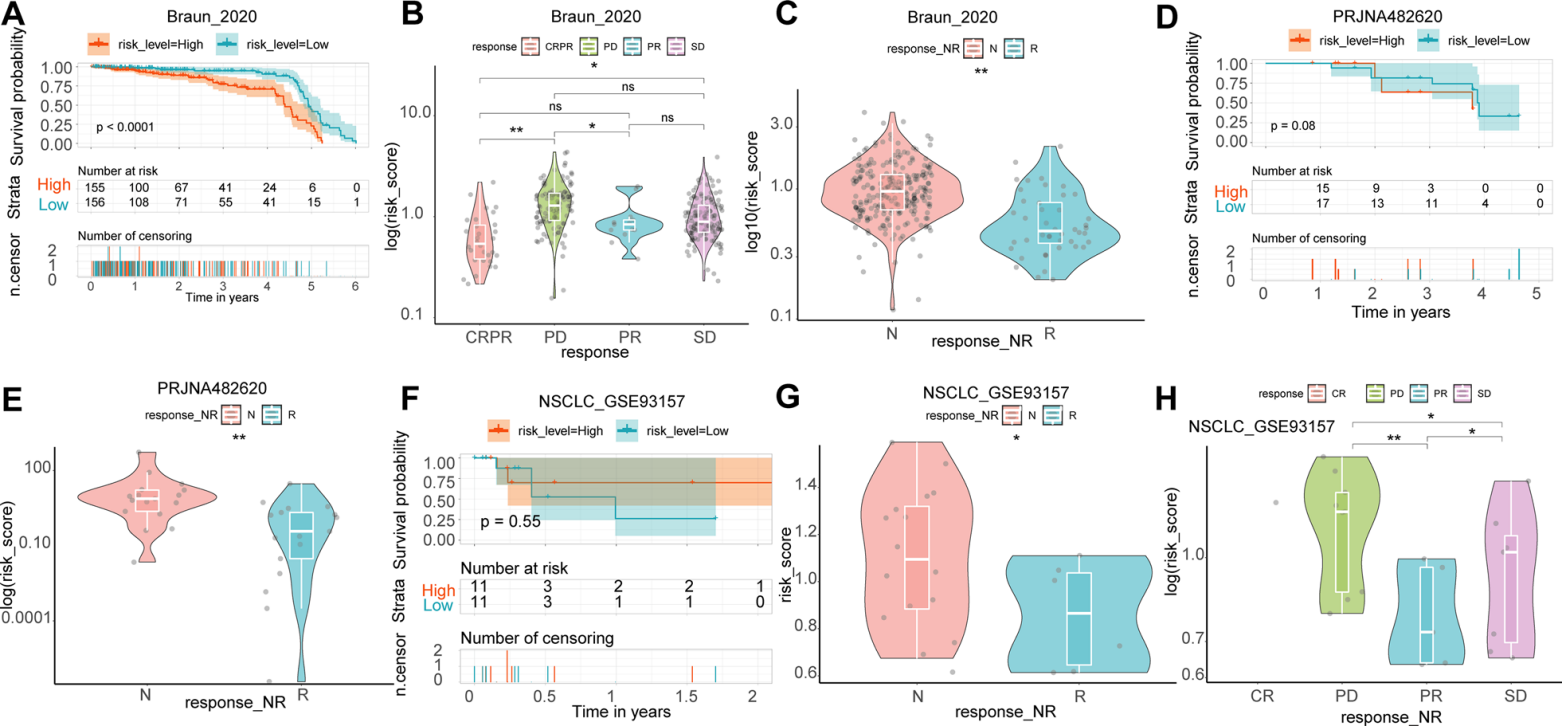
Validate the ability of CRSS to predict the effect of immunotherapy in the immunotherapy cohort of other tumors. KM analysis was used to compare the difference of immunotherapy effect between high and low CRRS groups in Braun_2020 (**A**), GBM-PRJNA482620 (**D**), and nonsqNSCLC-GSE93157 (**F**) dataset. Comparison of CRRS of other tumor patients in different remission states after immunotherapy in Braun_2020 (**B**) and nonsqNSCLC-GSE93157 (**H**) dataset. Comparison of CRRS of other tumor patients in different response states after immunotherapy in Braun_2020 (**C**), GBM-PRJNA482620 (**E**), and nonsqNSCLC-GSE93157 (**G**) dataset. *: P < 0.05; **: P < 0.01; ***: P < 0.001; ****: P < 0.0001

**Fig. 7:**
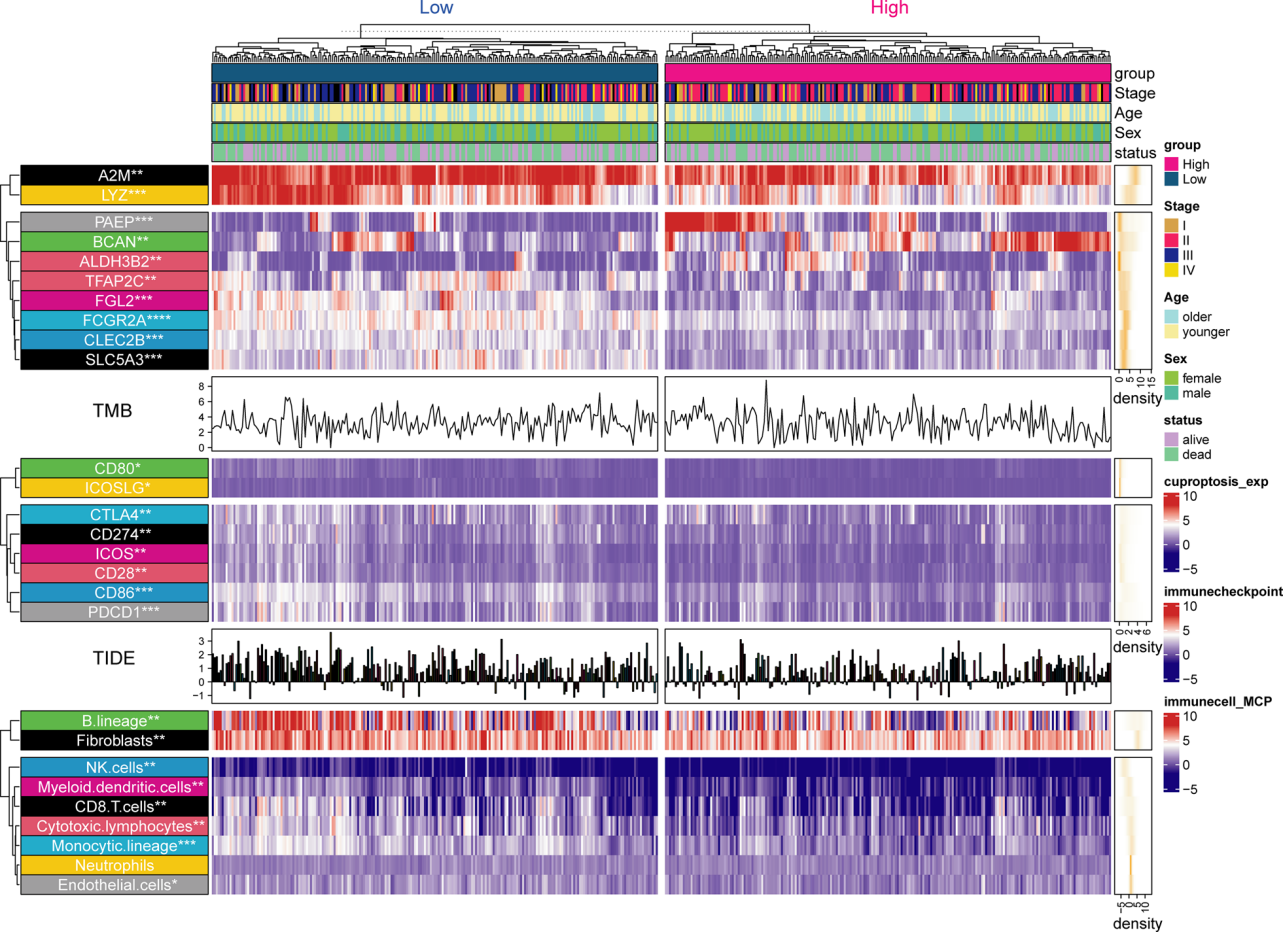
Landscape of the expression of significantly differentially expressed CRRS’ genes, TMB, TIDE score, Expression of immune checkpoints, and abundance of immune cell infiltration in the TCGA-SKCM cohort. The results were shown in the form of a complex heat map, in which the expression of significantly differentially expressed CRRS’ genes, Expression of immune checkpoints, and the abundance of immune cell infiltration predicted by MCP between the two groups was presented in the form of a heatmap. The TIDE score and TMB are displayed in the form of bar chart and density chart, respectively. The yellow density chart on the right side of the heatmap shows the average of each index. The statistical method of Mann- WhitneyU test was used to compare the two groups. *: P < 0.05; **: P < 0.01; ***: P < 0.001; ****: P < 0.0001

We validated the ability of CRSS to predict the effect of immunotherapy in eight cohorts of CM patients. In the phs000452 cohort (n = 153) containing complete clinical information on immunotherapy, the OS of CM patients with a high cuproptosis-related score was significantly better than CM patients with a low cuproptosis-related score (Fig. 5A). When comparing the CRRS of CM patients in different states during CM treatment, we found that CRRSs increased gradually from complete response (CR) to partial response (PR) to stable disease (SD) and then to progressive disease (PD) (Fig. 5B). In addition, CRRS in CM patients who responded to immunotherapy was significantly lower than in patients who did not respond to immunotherapy (Fig. 5C). Similar results from the PRJEB23709 (n = 91), GSE91061 (n = 109), GSE93157 (n = 25), GSE100797 (n = 25), GSE78220 (n = 28), GSE106128 (n = 47), and Nathanson_2017 (n = 24) datasets validated our conclusion (Fig. 5D–T). In addition, when CRSS was applied to other tumor immunotherapy cohorts such as renal cell carcinoma (Braun_2020, n = 311), glioblastoma (PRJNA482620, n = 34), and non-small cell lung cancer (nonsqNSCLC-GSE93157, n = 22), it also accurately predicted the effect of immunotherapy (Fig. 6A–H). Further, TMB in the high CRRS group was significantly lower than in the low CRRS group (Fig. 7). The TIDE analysis results showed that the TIDE score of the low CRRS group was significantly lower than in the high CRRS group, and immunotherapy response rate in the low CRRS group was significantly higher than in the high CRRS group (Fig. 7).

### Higher immune activity in the low CRRS group

First, we compared differences in immune checkpoint expression and immunocyte infiltration abundance between the high and low cuproptosis-related risk score groups. As shown in Fig. 7, the expression of eight common immune checkpoints including PDCD1, CD86, CD80, CD274, ICOS, ICOSLG, CTLA4, and CD28 were upregulated in the high CRRS group. MCP analysis suggested that the abundance of NK cells, monocyte lines, B cell lines, cytotoxic lymphocytes, and CD8 + T cells in patients with a low CRRS were significantly higher than in patients with a high CRRS (Fig. 7). In addition, we investigated the expression of 10 genes used to construct the CRSS between the two groups. The results showed that A2M, LYZ, TFAP2C, FGL2, FCGR2A, CLEC2B, and SLC5A3 were upregulated in the low CRRS group where PAEP, BCAN, and ALDH3B2 were downregulated in the low-rated group (Fig. 7). In addition, we used the online website GEPIA2 (http://gepia2.cancer-pku.cn/#index) [36] to analyze differences in expression of the 10 genes between the CM tissues from the TCGA database and normal skin tissues from the GTEx database. The results showed that compared with normal tissues, A2M, BCAN, FCGR2A, and LYZ were significantly upregulated and ALDH3B2, CLEC2B, and TFAP2C were significantly downregulated in CM tissues (Additional file 1: Fig. S7).

### Construction of the prognosis nomogram based on clinical features and CRSS

**Fig. 8:**
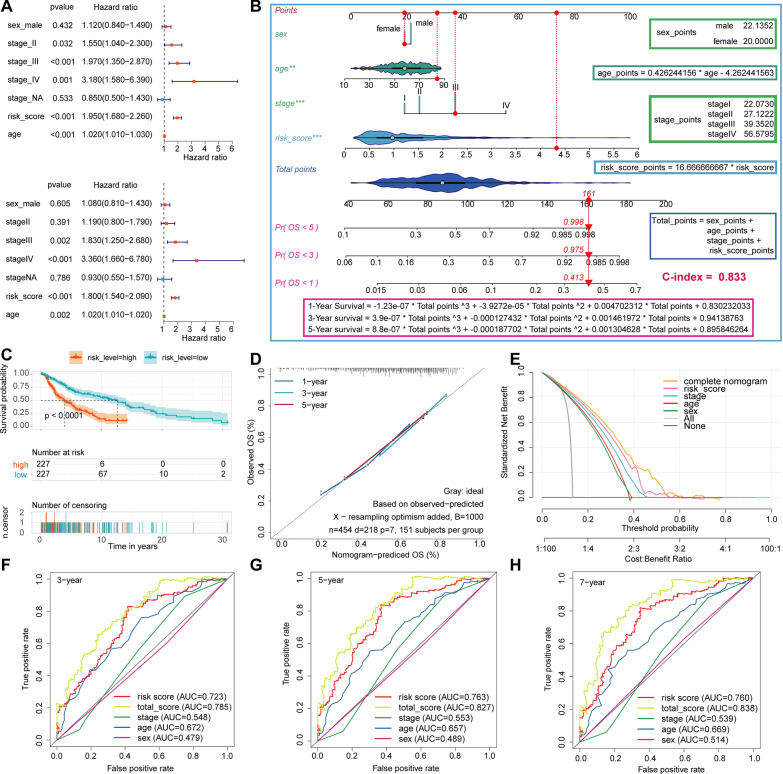
Construction of prognosis nomogram based on clinical features and CRSS. **A**. Univariate and multivariate COX regression analysis of CRSS and other clinical characteristics of all CM patients in the training cohort. **B**. Using CRSS and other clinical features of CM patients to construct prognostic Nomogram in the training cohort. The scoring function of each variable and the formula for calculating the survival rate are shown on the right and bottom of the graph, respectively. **C**. KM analysis was used to compare the difference of immunotherapy effect between high and low CRRS groups in training cohort. **D**. Calibration curve analysis validate the stability of the model. **E**. DCA evaluates the clinical practicability of the model and calculates and compares the clinical benefit rate of each model. **F**, **H**. Using ROC analysis to evaluate the ability and accuracy of the model to predict 3 -, 5-and 7-year OS in patients with CM

To use CRSS more effectively in predicting the prognosis of patients with CM, and more accurately stratify the risk of CM patients in the clinic, we included three clinical features (age, sex, and AJCC stage) as well as the use of CRSS to construct a prognostic nomogram model. Univariate (Fig. 8A, HR = 1.95, P < 0.001) and multivariate (Fig. 8A, HR = 1.80, P < 0.001) COX regression analyses showed that CRSS was an independent risk factor for patients with CM. Then, we constructed the nomogram model based on these four variables, quantified the score of each variable, and constructed the calculation formula of the total points and the calculation formula for survival probability (Fig. 8B). The specific formula is as follows:

$${\text{age}}_{{{\text{points}}}} = 0.426244156*age - 4.262441563;$$

$${\text{CRSS}}_{{{\text{points}}}} = 16.666666667*CRSS;$$

$${\text{Sex}}\;{\text{points}}:{\text{ sex of male}}_{{{\text{points}}}} = 22.13515;{\text{sex of female}}_{{{\text{points}}}} = 20.0;$$

$$\text{Stage}\;\text{points}:\text{ stage }{\text{I}}_{\text{point}}=22.072986,\text{ stage }{\text{II}}_{\text{point}}=27.122168,$$

$$\text{stage }{\text{III}}_{\text{point}}=39.351985, \text{stage }{\text{IV}}_{\text{point}}=56.579527$$

$${\text{Total}}_{\text{point}}={\text{age}}_{\text{points}}+{\text{CRSS}}_{\text{points}}+{\text{Sex}}_{\text{points}}+ {\text{Stage}}_{\text{points}}$$

$$1 - {\mathbf{Year}}\;{\mathbf{Survival}}_{{{\mathbf{rate}}}} = - 1.23{\text{e}}^{ - 7} {\text{* Total}}_{{{\text{point}}}}^{3} + - 3.9272{\text{e}}^{ - 5} {\text{* Total}}_{{{\text{point}}}}^{2} + 0.004702312{\text{ * Total}}_{{{\text{point}}}} + 0.830232033$$

$$3 - {\mathbf{Year}}\;{\mathbf{Survival}}_{{{\mathbf{rate}}}} = 3.9{\text{e}}^{ - 7} {\text{* points}}^{3} + - 0.000127432{\text{* points}}^{2} + 0.001461972{\text{ * points }} + 0.94138763$$

$$5 - {\mathbf{Year}}\;{\mathbf{Survival}}_{{{\mathbf{rate}}}} = 8.8{\text{e}}^{ - 7} {\text{* points}}^{3} + - 0.000187702{\text{* points}}^{2} + 0.001304628{\text{ * points }} + 0.895846264$$

The red line in Fig. 8B shows an example of our calculation using the above formula. Briefly, the ID of this sample was TCGA-BF-A3DL, an 84-year-old female CM patient with AJCC stage III; her CRRS was 4.3257325. According to the formula, her total points were 161, corresponding to the probability of an OS less than five years, three years, and one year of 0.998, 0.975, and 0.413, respectively. In reality, her OS was 2.107, which was very consistent with our prediction.

In addition, the C index of our model was as high as 0.833. Next, we validated the accuracy, stability, and clinical practicality of our model through KM, ROC, calibration curve analysis, and DCA. According to the coefficients of four variables in the multivariate COX regression analysis, we calculated the total risk score including four influencing factors (i.e., the total score). Taking the median of the total score as the cutoff value, all patients with CM were divided into two groups with high and low total scores. KM analysis showed that the OS of CM patients with a low total score was significantly better than that of CM patients with a high total score (Fig. 8C, log‐rank test P‐value < 0.0001). ROC analysis showed that the AUC values for 3-, 5-, and 7-year survival of CM patients predicted by the total score were 0.785, 0.827, and 0.838, respectively (Fig. 8F–G). In addition, the AUC value was higher than when each of the four variables was used alone to predict OS. The results from the calibration curve showed that the predicted values were consistent with the observed values for 1-, 3-, and 5-year OS (Fig. 8D). Finally, clinical decision curve analysis (DCA) showed that the clinical benefit rate of CRSS alone was significantly higher than that of AJCC stage. The clinical benefit rate of the model constructed by combining four variables was higher than that of each single variable (Fig. 8E). Based on the above results, we conclude that our prediction model has the ability to robustly and accurately predict prognosis.

### Validation of the prognostic model

To further validate the accuracy of our model, we applied the model to the validation cohort to test its applicability. Univariate (Additional file 1: Fig. S8A, HR = 2.34, P < 0.001) and multivariate (Additional file 1: Fig. S8A, HR = 1.94, P < 0.001) COX regression analyses validated that CRSS was an independent risk factor for patients with CM. In addition, we also used the DynNom package to validate the results of the nomogram. The results showed that 5-year survival rate decreased with increase in risk score in both stage I and stage IV (Additional file 1: Fig. S8B). KM analysis showed that in the validation cohort, the OS of CM patients with a high total score was significantly worse than CM patients with a low total score (Additional file 1: Fig. S8C, log‐rank test P‐value < 0.0001). ROC analysis showed that the AUC values for 3-, 5-, and 7-year survival in CM patients predicted by the total score were 0.863, 0.872, and 0.871, respectively (Additional file 1: Fig. S8F–G). The results of the calibration curve showed that the predicted values were consistent with the observed values for 1-, 3-, and 5-year OS (Additional file 1: Fig. S8D). Finally, clinical DCA showed that the clinical benefit rate of CRSS alone was significantly higher than AJCC stage. The clinical benefit rate of the model constructed by combining four variables was higher than that of each single variable alone (Additional file 1: Fig. S8E). Based on the above results, we conclude that our prediction model has the ability to robustly and accurately predict prognosis.

## Discussion

Immunotherapy is one of the most important treatments for patients with advanced CM. Immune activity and TME status of CM patients plays a decisive role in the effect of immunotherapy. Cuproptosis is a newly identified mode of cell death. Many studies have confirmed that CRGs are closely related to tumor immunotherapy and TME status. To date, there have been no studies focused on the relationship between cuproptosis and TME in CM, or the effect on immunotherapy.

In this study, we systematically divided TCGA-SKCM patients into two groups based on the 10 most classical CRGs using unsupervised cluster analysis. The immune score, the abundance of immune cell infiltration, the expression of immune checkpoints, TMB, and other TME indices in group A were significantly higher than in group B. In addition, the effect of immunotherapy and overall prognosis in group A were significantly better than in group B. We also evaluated the sensitivity of the two groups of CM patients to common immunotherapeutic drugs.

To explore the causes of the differences in prognosis and construct a risk prediction model, we performed difference analysis using univariate and multivariate COX regression analyses. We identified 10 genes that were most likely to lead to significant differences in immunotherapy efficacy and prognosis between the two groups. The CRSS composed of 10 the CRGs accurately stratified the risk of CM patients and showed that immune cell infiltration abundance, TMB, immunotherapy sensitivity, and immunotherapeutic effect in patients with high CRRS were significantly higher than in those with low CRRS. CRSS accurately predicted immunotherapeutic effect in 11 cohorts, including eight CM immunotherapy cohorts and three other tumor immunotherapy cohorts. In addition, the prognostic nomogram model constructed using CRSS and clinicopathological features was more accurate and stable than CRSS or AJCC stage alone.

C-Type Lectin Domain Family 2 Member B (CLEC2B) encodes a member of the C-type lectin/C-type lectin-like domain (CTL/CTLD) superfamily. Members of this family share common protein folding and have a variety of functions such as cell adhesion, intercellular signal transduction, glycoprotein conversion, and roles in inflammation and immune responses. CLEC2B encodes a type 2 transmembrane protein that functions as a cell activation antigen [37]. However, the research on CLEC2B and CM is currently lacking. In this study, compared with normal tissues and CM patients with low CRRS, CLEC2B was significantly downregulated in CM patients and CM patients with high CRRS. Moreover, univariate (HR = 0.690) and multivariate (HR = 0.810) COX regression analyses showed that CLEC2B was an independent protective factor for patients with CM. Therefore, CLEC2B may inhibit the occurrence and development of CM by enhancing immune surveillance.

Fc Gamma Receptor Iia (FCGR2A) encodes a member of the immunoglobulin Fc receptor gene family found on the surface of many immunoreactive cells. The protein encoded by this gene is a cell surface receptor present on phagocytes such as macrophages and neutrophils and is involved in the phagocytosis and clearance of immune complexes. Following binding to IgG, FCGR2A initiates cellular responses against pathogens and soluble antigens. FCGR2A also promotes phagocytosis of opsonized antigens [38]. Mutations or deletions of FCGR2A can lead to significant resistance to immunotherapy in a variety of tumor types including colon cancer, breast cancer, and leukemia [39–41]. The protein encoded by Alpha-2-Macroglobulin (A2M) is a protease inhibitor and cytokine transporter. A2M uses decoy and trap mechanisms to inhibit broad-spectrum proteases including trypsin, thrombin, and collagenase, and inhibits inflammatory cytokines, thereby disrupting the inflammatory cascade [42]. Lindner et al. found that A2M inhibits the malignant properties of astrocytoma cells by impeding beta-catenin signaling [43]. There are currently no mechanistic studies exploring the link between A2M, FCGR2A, and CM. In the present study, A2M and FCGR2A were simultaneously upregulated in CM and downregulated in CM patients with a high CRRS relative to patients with a low CRRS. Further, univariate and multivariate COX regression analyses suggested that both A2M (HR = 0.860) and FCGR2A (HR = 0.820) were independent protective factors in CM patients. Therefore, we hypothesize that FCGR2A and A2M may enhance the efficacy of immunotherapy and improve prognosis by enhancing immune activity and immune lethality in CM patients.

Brevican (BCAN) encodes a member of the chondroitin sulfate proteoglycan glycan family that is specifically expressed in the central nervous system. This protein is regulated during development and may play a role in cell adhesion. BCAN is highly expressed in glioma and may promote the growth and cell motility of brain tumor cells [44, 45]. However, there is no reported research on BCAN and CM. In our study, the expression of BCAN in the tumor and high CRRS groups was significantly higher than in normal tissues and the low CRRS group. In addition, univariate (HR = 1.09) and multivariate (HR = 1.06) COX regression analyses showed that BCAN is an independent risk factor in patients with CM. Therefore, we speculate that BCAN may promote the development of CM by affecting cell adhesion.

This study had some limitations. First, the data were retrospective, and a prospective cohort would be needed to validate our model. Second, the expression of each gene in our model was based on skin tissues. Development of biomarkers based on urine or blood samples would be more appropriate for clinical application. Finally, future studies should include in *vivo* and in *vitro* experiments to verify the specific mechanisms of each molecule in CM.

In the future, we will first retrospectively collect biological samples and clinical data of past and prospective CM patients in our hospital, and construct an external validation cohort to validate the accuracy of our signature in predicting the effect of immunotherapy, as well as the accuracy of our model in predicting the prognosis of CM patients. Then, biological specimens and detailed clinical data of CM patients admitted to our hospital will be prospectively collected under the condition of ethical review and informed consent signed by patients. The required sample size was calculated according to the effect value, test level (*α*) and test efficacy (*1-β*) equivalence of the previous retrospective study results. The experimental group and the control group were matched by age, sex, clinical staging, and other basic characteristics. A 5-year follow-up will be conducted to record the status of CM patients after immunotherapy, as well as the time to death. We wound calculate the accuracy of our signature in predicting immunotherapy effect in CM patients and the ability of model scores to predict prognosis in CM patients at a follow-up time of 6 months, 1 year, 3 years, and 5 years.

Overall, we first attempted to developed a new cuproptosis-related scoring system that can stratify the risk of CM patients and predict their prognosis, thus potentially affecting immunotherapeutic choices. Moreover, we constructed a prognostic nomogram model to more efficiently guide clinical decision making.

## Supplementary Information

**Additional file 1: Table S1.** Basic information of the datasets included in this study. **Table S2.** Basic information of immuntherapy datasets included in this study. **Figure S1.** The flowchart of the study. **Figure S2.** Unsupervised clustering for CRGs. **Figure S3.** The difference of immune score between group A and B. Figure S4. GSEA. Figure S5. The distribution of CRRS, alive status, and gene expression panel. **Figure S6.** Validation of the prognostic value of CRSS for CM. **Figure S7.** The difference in the expression of CRGs molecules between CM tissues and normal skin tissues. **Figure S8.** Validation of the model in the validation cohort.

## Data Availability

The data from the TCGA and GEO data sets in this study are publicly available.
