# Supplementary material for: Leveraging a cuproptosis-based signature to predict the prognosis and drug sensitivity of cutaneous melanoma
Source: J Transl Med. 2023 Jan 30;21:57. doi: 10.1186/s12967-023-03891-4 (PMC9885583; doi:10.1186/s12967-023-03891-4)
Supplement: Supplementary file 1 — Additional file 1: Table S1. Basic information of the datasets included in this study. Table S2. Basic information of immuntherapy datasets included in this study. Figure S1. The flowchart of the study. Figure S2. Unsupervised clustering for CRGs. Figure S3. The difference of immune score between group A and B. Figure S4. GSEA. Figure S5. The distribution of CRRS, alive status, and gene expression panel. Figure S6. Validation of the prognostic value of CRSS for CM. Figure S7. The difference in the expression of CRGs molecules between CM tissues and normal skin tissues. Figure S8. Validation of the model in the validation cohort. [file 12967_2023_3891_MOESM1_ESM.docx]

**Additional tables**

Table-S1: Basic information of datasets included in this study.

| **Accession number /Source** | **Platform** | **Number of  patients** | **Stage** | **Survival data** |
| --- | --- | --- | --- | --- |
| GEO: GSE22153 | GPL6102 | 57 | III:3 IV:54 | OS |
| GEO: GSE22154 | GPL6947 | 22 | IV:22 | OS |
| GEO: GSE46517 | GPL96 | 121 | I/II:27 III/IV:55 | OS |
| GEO: GSE54467 | GPL6884 | 79 | I/II:58 III/IV:24 | OS |
| GEO: GSE65904 | GPL10558 | 214 | NA | DFS/DMFS |
| GEO: GSE15605 | GPL570 | 74 | NA | NA |
| TCGA: SKCM | Illumina RNAseq | 469 | I/II:147 III/IV:240 | OS/DFS |

**Table S2: Basic Information of immunotherapy datasets included in this study.**

| dataset id | cancer type | PMID | Therapy | dataset size | responder-number | non-responder number |
| --- | --- | --- | --- | --- | --- | --- |
| GBM-PRJNA482620 | Glioblastoma | 30996326 | anti-PD-1 | 34 | 17 | 17 |
| Melanoma-GSE100797 | Melanoma | 29170503 | ACT | 25 | 10 | 15 |
| Melanoma-GSE106128 | Melanoma | 29682201 | DCs_treated | 47 | 21 | 14 |
| Melanoma-GSE78220 | Melanoma | 28129544 | anti-PD-1 | 28 | 15 | 13 |
| Melanoma-GSE91061 | Melanoma | 29033130 | anti-PD-1 | 109 | 20 | 78 |
| Melanoma-GSE93157 | Melanoma | 28487385 | anti-PD-1 | 25 | 9 | 16 |
| Melanoma-Nathanson_2017 | Melanoma | 27956380 | anti-CTLA-4 | 24 | 8 | 16 |
| Melanoma-phs000452 | Melanoma | 26359337 | anti-PD-1 | 153 | 63 | 90 |
| Melanoma-PRJEB23709 | Melanoma | 30753825 | anti-PD-1 and anti-CTLA-4+anti-PD-1 | 91 | 49 | 42 |
| nonsqNSCLC-GSE93157 | Nonsmall-cell lung cancer | 28487385 | anti-PD-1 | 22 | 6 | 16 |
| RCC-Braun_2020 | Renal cell carcinoma | 32472114 | anti-PD-1 and EVEROLIMUS | 311 | 44 | 237 |

**Supplementary Figures**


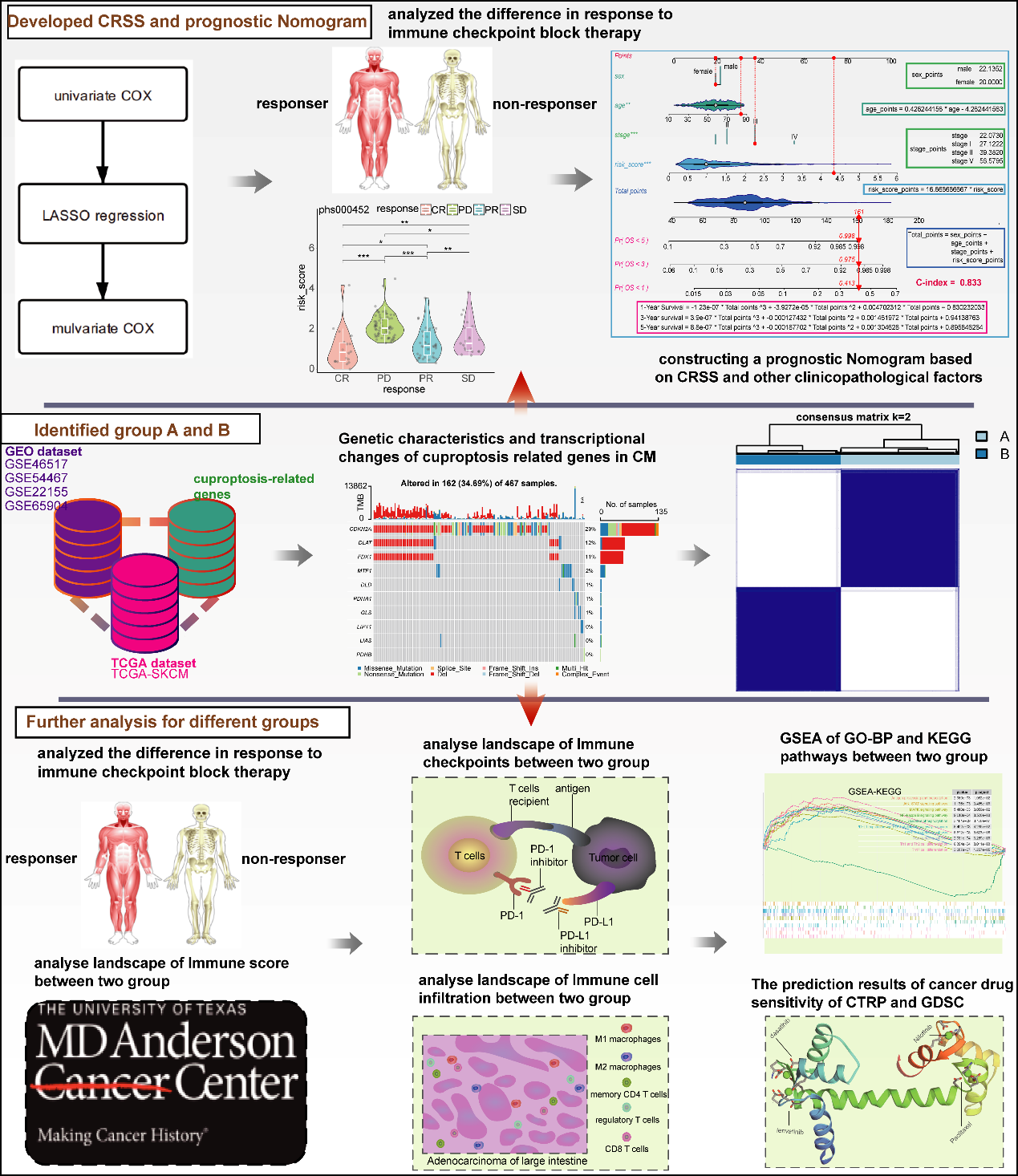


**Figure S1. The flowchart of the study.**


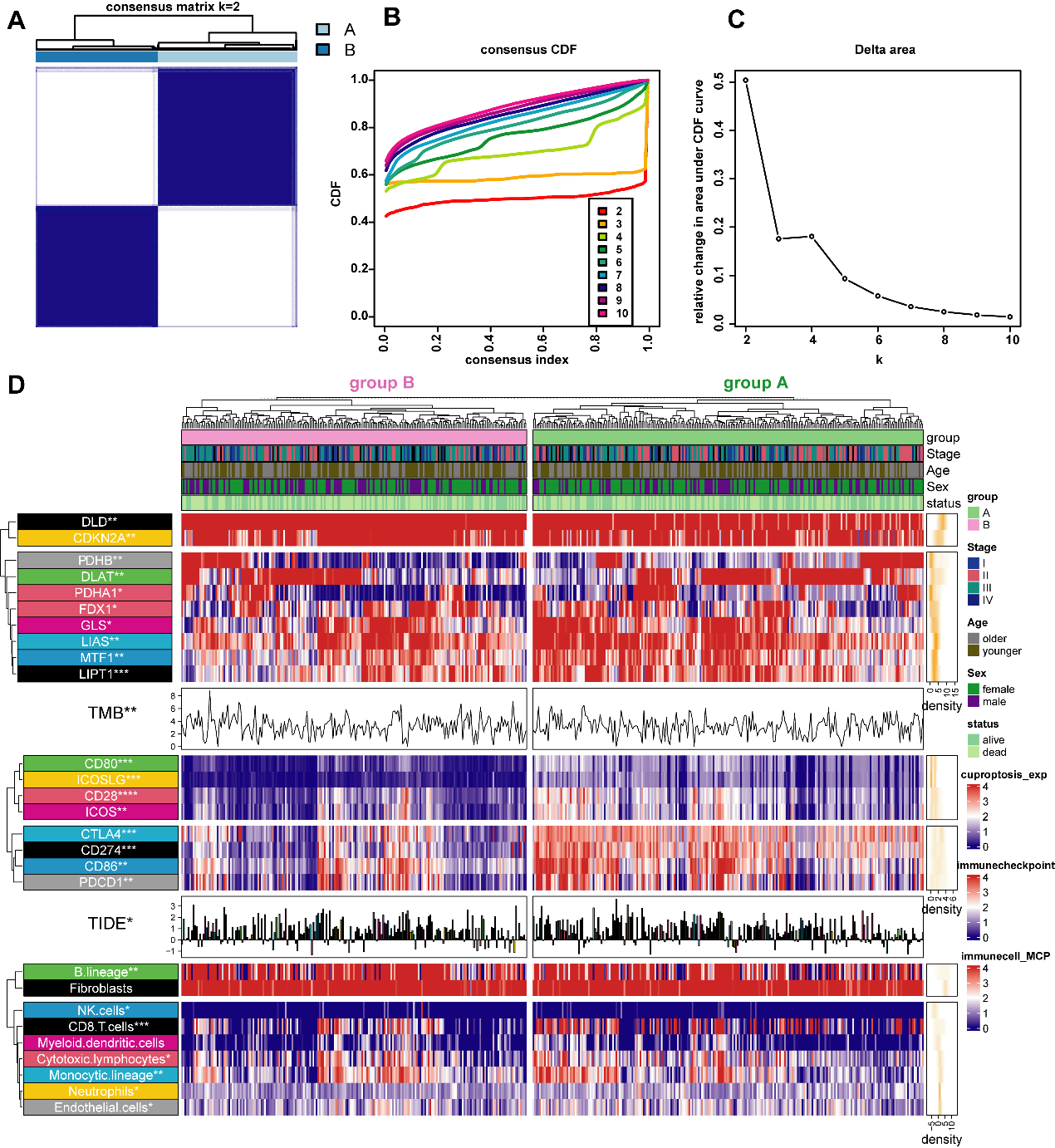


**Figure S2. Unsupervised clustering for CRGs. (A-C)** Classification of TCGA-SKCM into two groups. When the cumulative distribution function (CDF) reaches the approximate maximum value, the cluster analysis result is the most reliable. Typically, a K value with small decline in the slope of the CDF is used. **(D)** Landscape of the expression of significantly differentially expressed CRGs, TMB, TIDE score, Expression of immune checkpoints, and abundance of immune cell infiltration in the TCGA-SKCM cohort. The results were shown in the form of a complex heat map, in which the expression of significantly differentially expressed CRGs, Expression of immune checkpoints, and the abundance of immune cell infiltration predicted by MCP between the two groups was presented in the form of a heatmap. The TIDE score and TMB are displayed in the form of bar chart and density chart, respectively. The yellow density chart on the right side of the heatmap shows the average of each index. The statistical method of Mann- WhitneyU test was used to compare the two groups. *: P < 0.05; **: P < 0.01; ***: P < 0.001; ****: P < 0.0001.


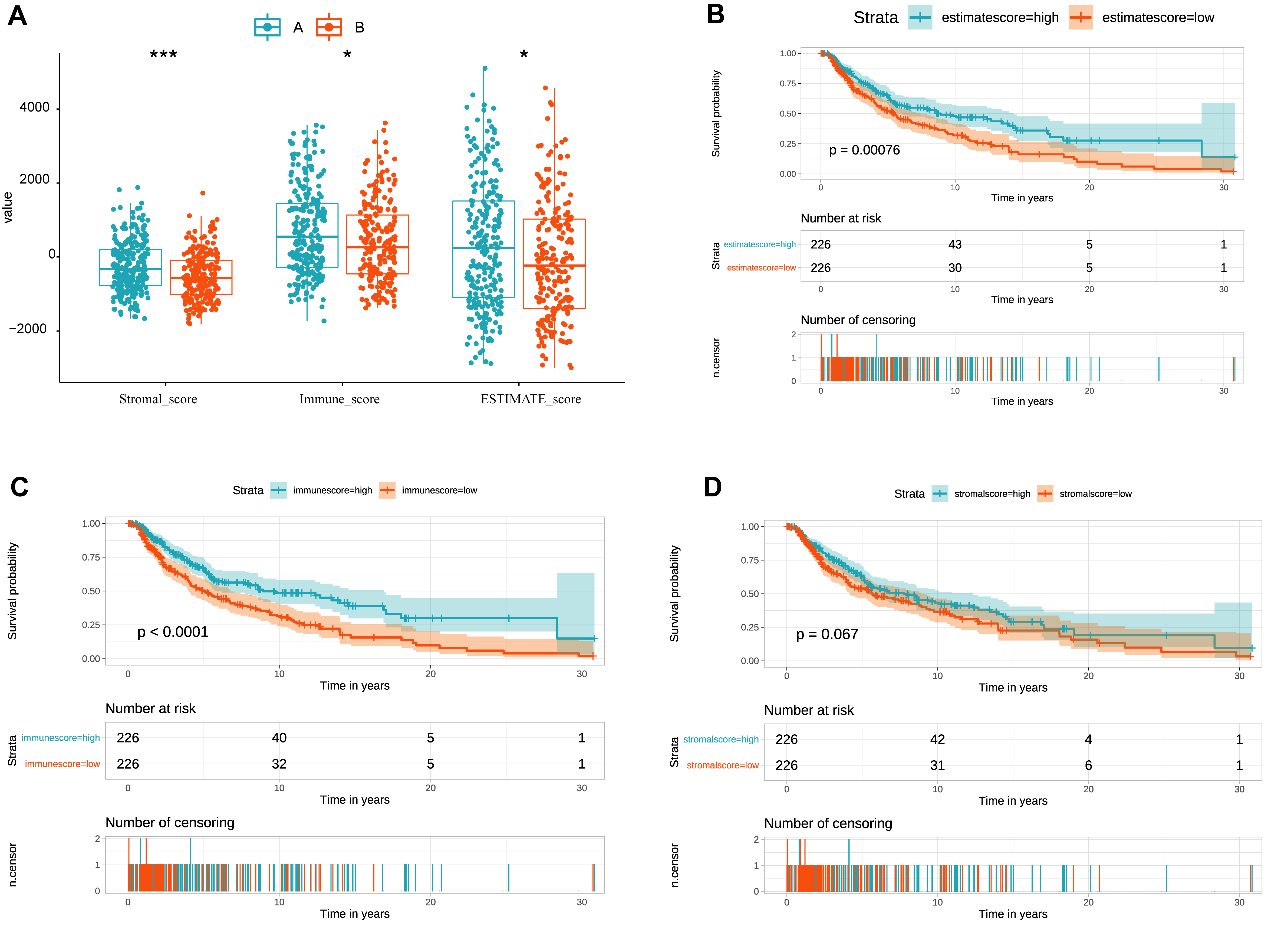


**Figure S3.** The difference of immune score between group A and B. Different immune and stromal scores between the two groups (**A**). Kaplan–Meier analysis of the CM data in the TCGA cohorts showed that OS was significantly shorter in patients with low stromal, immune, and ESTIMATE scores (**B, C, and D**). *: P < 0.05; **: P < 0.01; ***: P < 0.001; ****: P < 0.0001.


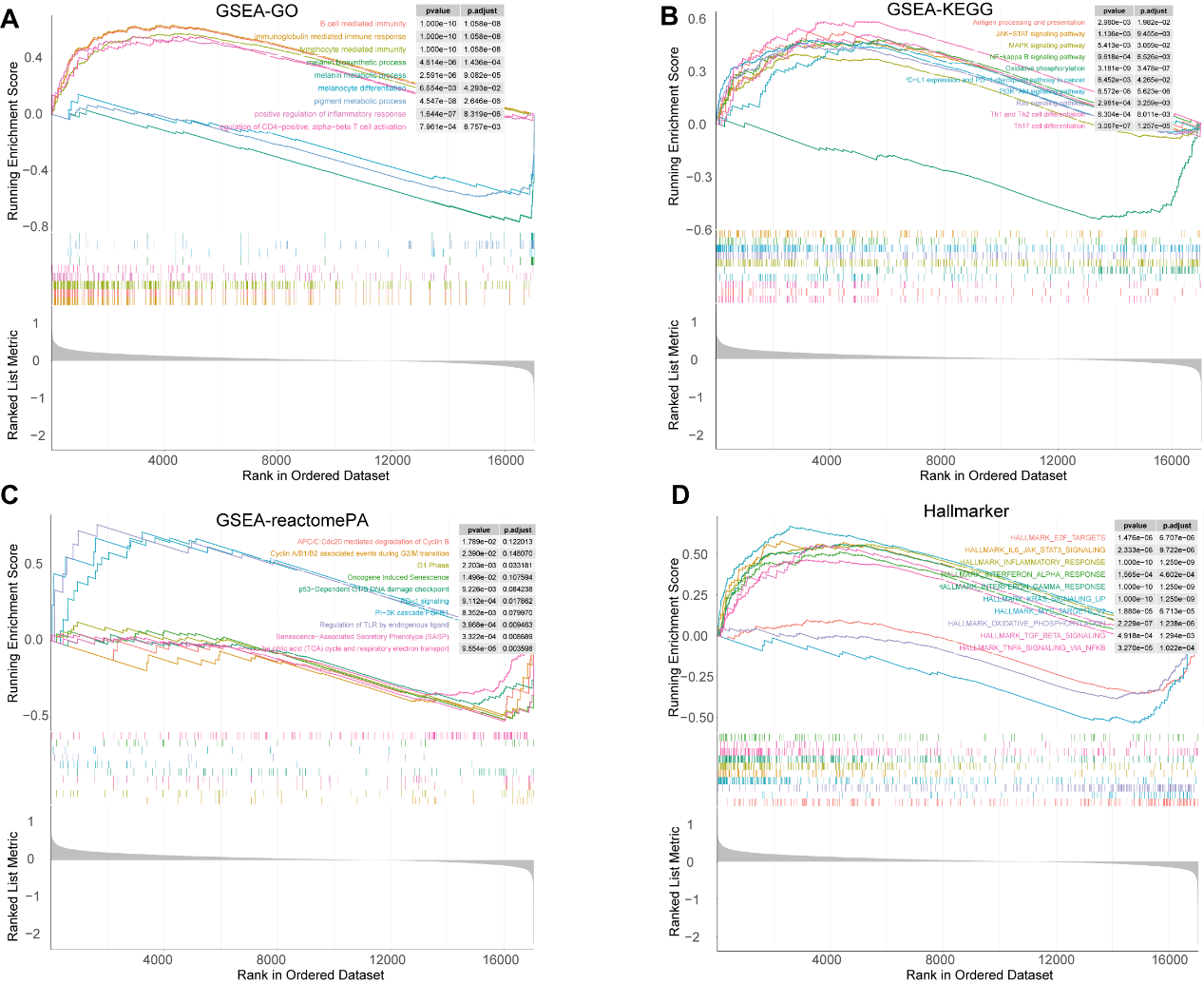


**Figure S4.** **GSEA.** the results of GSEA-GOBP, GSEA-KEGG, GSEA-reactomePA, and GSEA-hallmarker. ES, enrichment score; NES, normalized enrichment score; P, P-value; FDR, adjusted P-value.


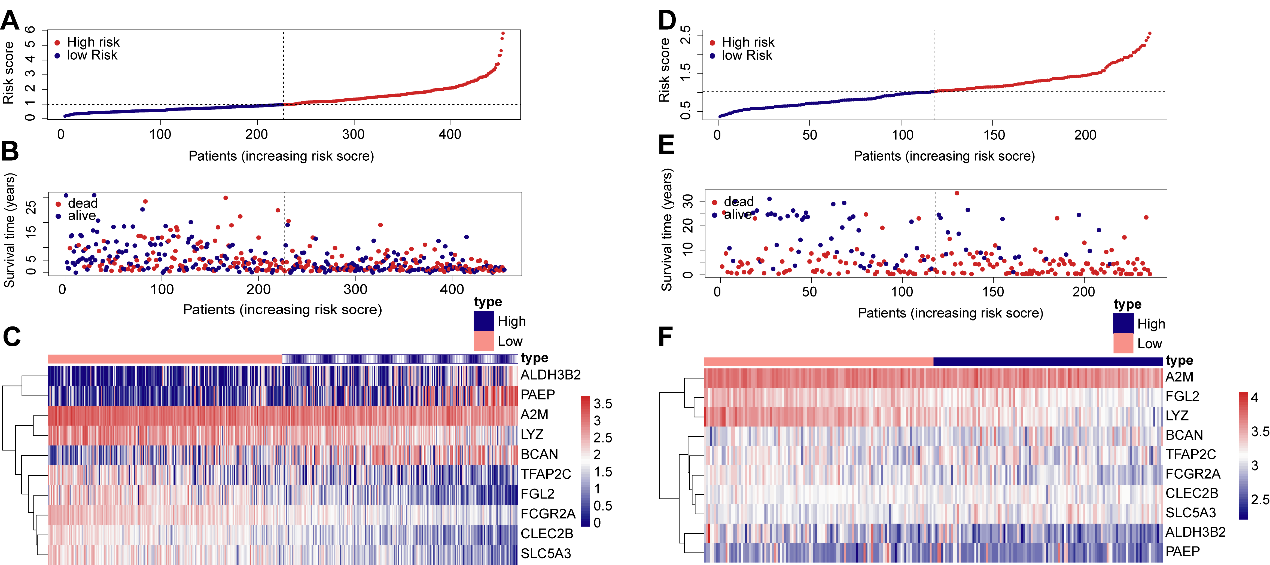


**Figure S5**. **The distribution of CRRS, alive status, and gene expression panel.** Correlation between CRGs and the OS of patients in the training (**A-C**) and validation (**D-F**) cohort. The distribution of CRRS (upper), survival time (middle), and CRGS expression levels (lower). The black dotted lines represent the median CRRS cut-off dividing patients into low- and high- CRRS groups. The red dots and lines represent the patients in the high-CRRS groups. The green dots and lines represent the patients in the low-CRRS groups.


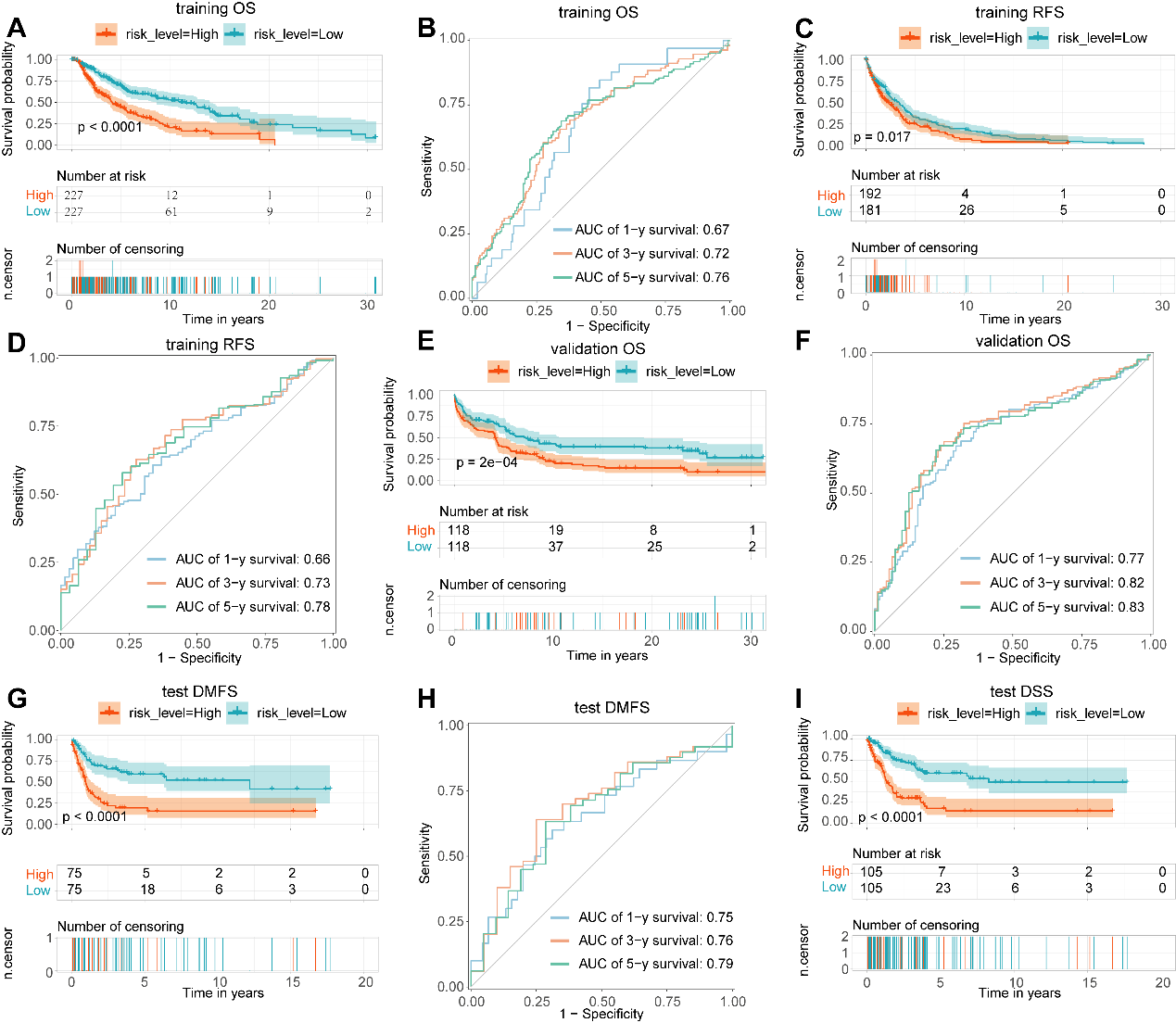


**Figure S6**. **Validation of the prognostic value of CRSS for CM**. In the TCGA cohort, the patients in the high-CRRS group had significantly worse OS and RFS than those in the low- CRRS group (**A and C**). Similar results can be observed in the validation cohort (**E**). In addition, ROC curve analysis was implemented to determine whether survival predictions made with the CRSS were accurate in the TCGA and validation cohorts (**B, D, and F**). The Kaplan-Meier curves for DSS and DMFS suggested that the patients with high- CRRS had significantly worse DSS and DMFS than those with low- CRRS (**G and I**). In addition, the results showed that the AUC for 5-year DMFS reached 0.79 (**H**).


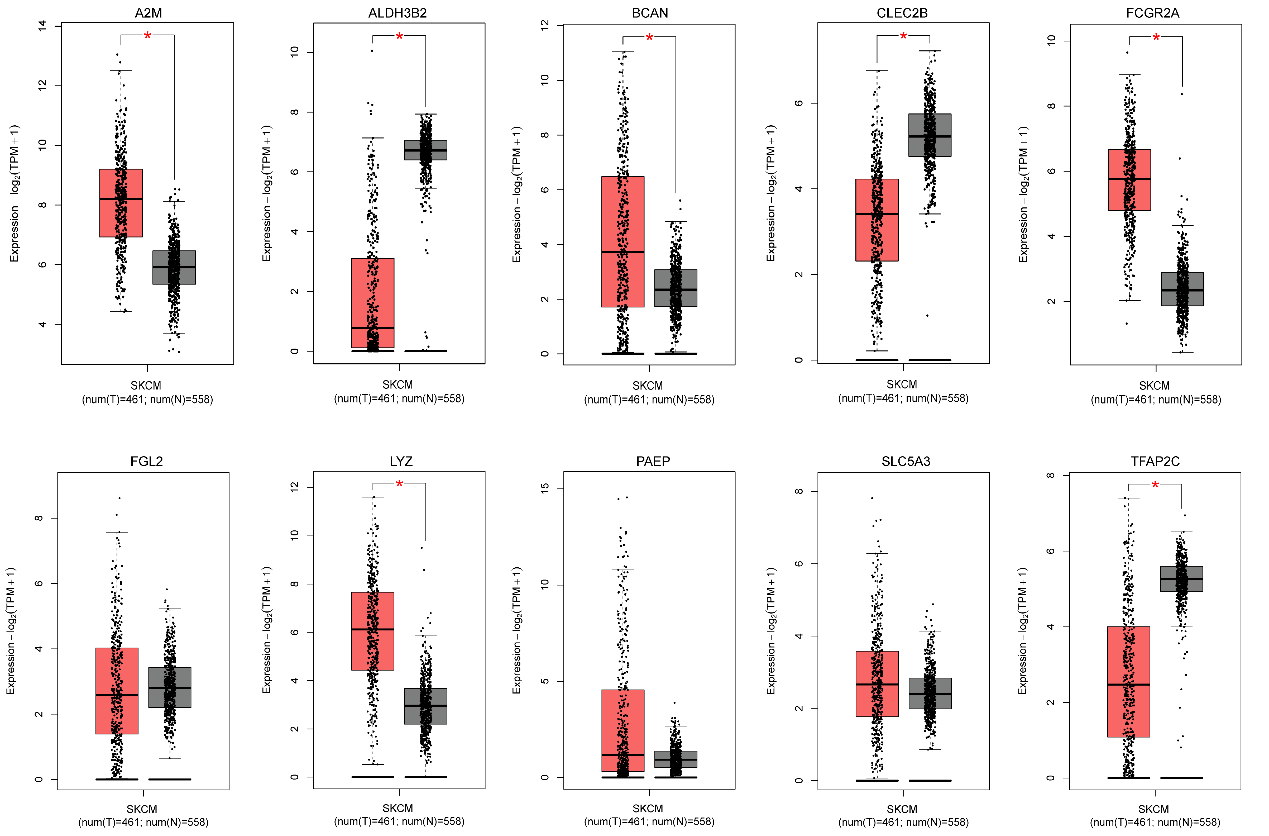


**Figure S7. The difference in the expression of** **CRGs molecules between CM tissues and normal skin tissues.** The difference in the expression of CRGs molecules between CM tissues in TCGA and normal skin tissues in GTEx. Red represents tumor tissue; gray represents normal tissue.


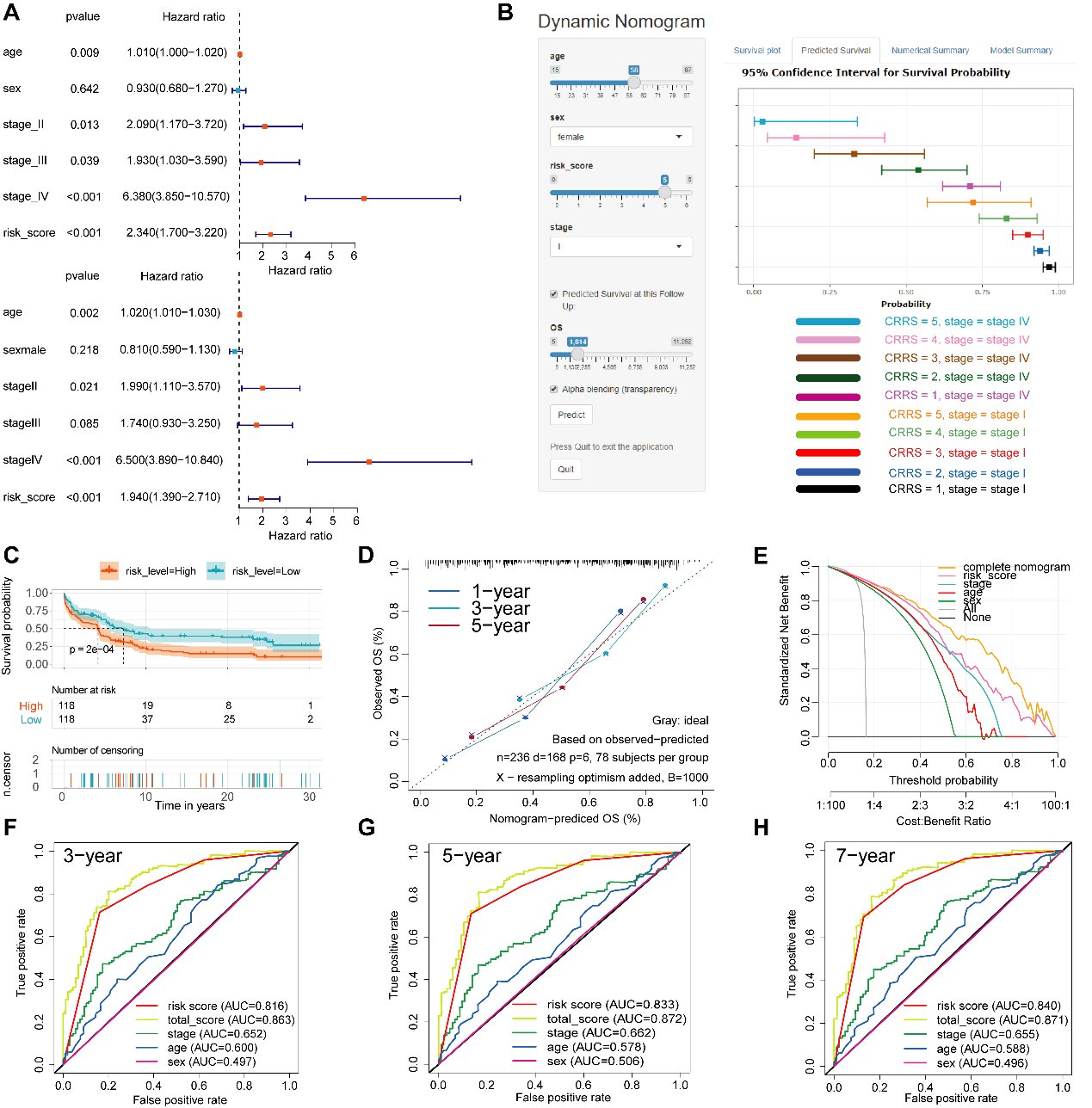


**Figure S8**. **Validation of the model in the validation cohort.** The univariate and multivariate Cox regression model (**A**). We use DynNom package to validate the results of nomogram. The results show that the 5-year survival rate decreases with the increase of risk score in both stage I and stage IV (**B).** Kaplan-Meier analysis showed that patients with a high-CRRS had an obviously worse OS than patients with a low-CRRS (**C**). According to the calibration curve, predictive values were consistent with observed values considering the probabilities of 1-year, 3-year, and 5-year OS **(D**). Finally, clinical decision analysis (DCA) shows that the clinical benefit rate of the model without stage alone is higher than that of the model without CRSS alone **(E**). The AUC values for 3-, 5-, and 7-year survival using the predictive nomogram reached 0.863, 0.872, and 0.871, respectively (**F-H**).
